# Diagnosis of Autism Spectrum Disorders Using Multi-Level High-Order Functional Networks Derived From Resting-State Functional MRI

**DOI:** 10.3389/fnhum.2018.00184

**Published:** 2018-05-14

**Authors:** Feng Zhao, Han Zhang, Islem Rekik, Zhiyong An, Dinggang Shen

**Affiliations:** ^1^School of Computer Science and Technology, Shandong Technology and Business University, Yantai, China; ^2^Department of Radiology and BRIC, University of North Carolina, Chapel Hill, NC, United States; ^3^BASIRA Lab, CVIP Group, Computing, School of Science and Engineering, University of Dundee, Dundee, United Kingdom; ^4^Department of Brain and Cognitive Engineering, Korea University, Seoul, South Korea

**Keywords:** autism spectrum disorder, high-order functional connectivity, brain network, resting-state fMRI, learning-based classification

## Abstract

Functional brain networks derived from resting-state functional magnetic resonance imaging (rs-fMRI) have been widely used for Autism Spectrum Disorder (ASD) diagnosis. Typically, these networks are constructed by calculating functional connectivity (FC) between any pair of brain regions of interest (ROIs), i.e., using Pearson's correlation between rs-fMRI time series. However, this can only be called as a *low-order* representation of the functional interaction, because the relationship is investigated just between two ROIs. Brain disorders might not only affect low-order FC, but also *high-order* FC, i.e., the higher-level relationship among multiple brain regions, which might be more crucial for diagnosis. To comprehensively characterize such relationship for better diagnosis of ASD, we propose a multi-level, high-order FC network representation that can nicely capture complex interactions among brain regions. Then, we design a feature selection method to identify those discriminative multi-level, high-order FC features for ASD diagnosis. Finally, we design an ensemble classifier with multiple linear SVMs, each trained on a specific level of FC networks, for boosting the final classification accuracy. Experimental results show that the integration of both low-order and first-level high-order FC networks achieves the best ASD diagnostic accuracy (81%). We further investigated those selected discriminative low-order and high-order FC features and found that the high-order FC features can provide complementary information to the low-order FC features in the ASD diagnosis.

## Introduction

Autism spectrum disorder (ASD) is a prevalent and highly heterogeneous childhood neurodevelopmental disease. It impairs children's social interaction, communication, and many other behavioral and cognitive functions in varying degrees (Ecker et al., 2010). According to the latest report released by the Centers for Disease Control and Prevention^1^, one out of 68 American children was affected by some form of ASD, an increase of 78% compared with the past decade. Accurate early diagnosis and timely intervention, especially for the infants under 12-month-old, may tremendously improve the outcome (Wolff et al., 2012; Jin et al., 2015; Zwaigenbaum et al., 2015). However, ASD is a very complex and highly heterogeneous disorder, involving many higher-level brain functions and even whole-brain structures and functions, which makes the diagnosis very challenging. To help tackle this challenge, several neuroimaging studies have used different non-invasive brain imaging modalities (Anagnostou and Taylor, 2011; Zhao et al., 2017), including structural magnetic resonance imaging (sMRI) (Wee et al., 2014a), electroencephalogram (EEG) (Duffy and Als, 2012), and diffusion tensor imaging (DTI) (Ingalhalikar et al., 2011; Gopikrishna et al., 2013), for developing computer-aided ASD diagnosis tools.

Recently, resting-state functional magnetic resonance imaging (rs-fMRI), which uses blood-oxygenation-level-dependent (BOLD) signals as a neurophysiological index to probe brain activity, has been applied to the diagnosis of ASD (Plitt et al., 2014; Price et al., 2014; Ha et al., 2015). Sensitive to the spontaneous and intrinsic neural activity, the BOLD signals can be used as effective and non-invasive measures to investigate neuropathological substrates of many neurological and psychiatric disorders at a whole-brain system level (Asghar et al., 2011; Keith et al., 2015). In particular, functional connectivity (FC), defined as the temporal correlation of the BOLD signals of different brain regions, reflect the close interactions of multiple brain regions that could be structurally segregated. In previous studies, many FC modeling methods have been proposed to construct brain functional networks, including Pearson's correlation, partial correlation, and sparse representation (Dijk et al., 2010; Wee et al., 2014b; Biao et al., 2016). However, most existing studies used Pearson's correlation for measuring FC due to its simplicity (Wee et al., 2012; Jie et al., 2014). However, the Pearson's correlation based FC networks can only capture the low-order functional relationship between two brain regions. This type of low-order FC networks may overlook more complex, high-order relationship that could be also altered in ASD children; thus, the use of additional high-order relationship may further help ASD diagnosis. Note that the high-order FC could capture the interaction among multiple brain regions, rather than simple pair-wise relationship. To date, several methods for constructing high-order FC networks have been developed (Chen et al., 2016; Wee et al., 2016; Zhang et al., 2016, 2017a,b; Zhou et al., 2018). For example, Chen et al. (2016) used sliding window approach to derive dynamic FC (time-varying FC) and then conducted additional round of Pearson's correlations (“correlation's correlation”) between each pair of dynamic FC time series to build a high-order FC network. A more neurobiologically intuitive high-order FC method was proposed by Zhang et al. (2016) for more sensitive early Alzheimer's disease detection and has been adopted in other studies (Zhang et al., 2017a,b; Zhou et al., 2018). This method also uses “correlation's correlation,” where the first round of correlation analysis generates regional FC topographical profiles (the FCs between one region to all other regions), which are further correlated between each pair of regions. In this way, the high-order FC represents similarity of FC topographical profiles, which supplements the traditional, BOLD-signal-synchronization-based low-order FC (Zhang et al., 2016). For more details, please refer to some previous methodological papers and clinical application papers (Chen et al., 2016; Wee et al., 2016; Zhang et al., 2016, 2017a,b; Zhou et al., 2018).

To the best of our knowledge, very few studies have used high-order FC for ASD children diagnosis. We hypothesize that brain networks in ASD children could be altered due to miswiring during abnormal development. Such miswiring could affect both low-order FC and high-order FC. Similar to the hypothesis behind Alzheimer's disease studies using high-order FC (Chen et al., 2016; Wee et al., 2016; Zhang et al., 2016, 2017b; Zhou et al., 2018), we propose that the high-order FC could be also affected in ASD and thus can be used as effective biomarkers for ASD diagnosis. There are two types of high-order FC methods previously proposed (Hansen et al., 2015; Chen et al., 2016; Wee et al., 2016; Zhang et al., 2016, 2017a,b; Glomb et al., 2017; Zhou et al., 2018). The first type of methods applied a second round of Pearson's correlation on the dynamic FC time series (Chen et al., 2016; Wee et al., 2016), but the neurological significance of the time-varying FC is still unclear and it could cause dramatically increased feature dimensionality (Zhang et al., 2016, 2017a), which could affect the robustness of classification model. The second type of methods is more straightforward (Zhang et al., 2016, 2017a,b; Zhou et al., 2018), by first calculating regional low-order FC topographical profiles (each characterizing the FC between one brain region and all other brain regions) and then using them as regional features to further compute another level of Pearson's correlation between any pair of brain regions (i.e., “correlation of correlations”). This kind of high-order FC networks could carry complementary information to the traditional low-order FC networks, and could be jointly used for improving ASD diagnosis. Theoretically, by repeating such a “correlation of correlations” analysis iteratively, one can generate many *higher*-*order* FC networks, each of which is derived from a precedent level of high-order FC network by computing the next higher level of correlations. Thus, it is of scientific and clinical importance to investigate (1) whether ASD diagnosis can benefit from high-order functional networks, and (2) to what extend integrating different levels of FC networks could improve the accuracy of ASD diagnosis.

**Figure 1:**
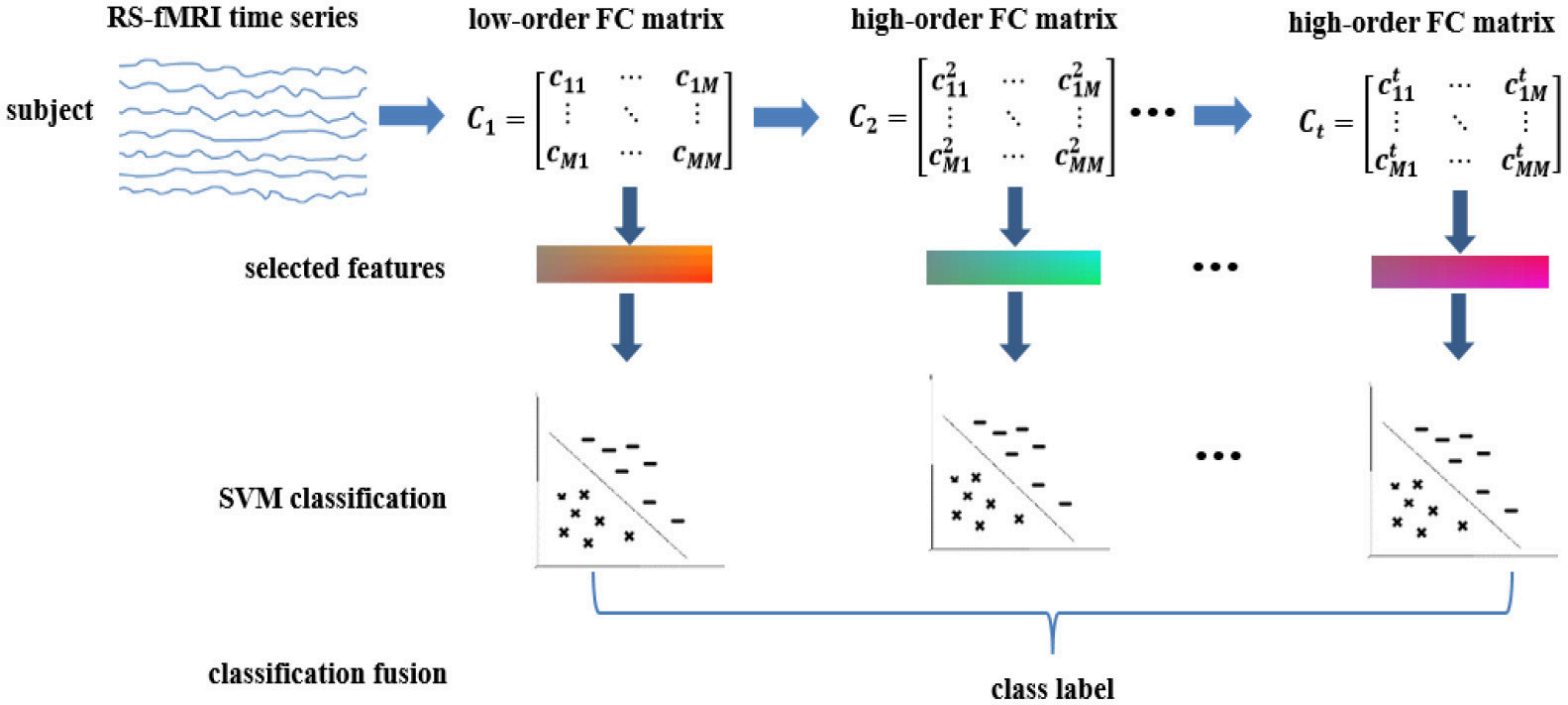
Overview of the proposed multi-level high-order functional connectivity classification framework for ASD diagnosis.

To explore these hypotheses, we extend our previous works on high-order FC by proposing high(er)-order brain functional network representations at multiple levels. We then use these multi-level FC networks (with different levels of functional interactions) for a joint and better ASD diagnosis. Furthermore, we devise a generalized, multi-level high(er)-order brain networks based classification framework, which includes an ensemble of multiple classifiers, each trained using a specific level of high(er)-order FC network to capture level-specific diagnostic information. We apply our new framework to the Autism Brain Imaging Data Exchange (ABIDE) database for individual-based classification between ASD children and normal controls (NC). Figure 1 shows the pipeline of the proposed classification framework, which mainly includes the following four steps:

1. Low-order FC network (*LON*) construction. We first estimate low-order FC network from the raw rs-fMRI time series. Each low-order network is represented as a correlation matrix.
2. Multi-level high-order FC network construction. We construct the first-level of high-order FC network (represented by an “*HON-1*” matrix), with each element as the Pearson's correlation coefficient between two associated low-order FC profiles from two corresponding brain regions. We iteratively derive the second-, third- and higher-levels of higher-order networks (i.e., *HON-2, HON-3*, and so on) by using their respective previous level of high(er)-order FC profiles.
3. LASSO-based feature selection. We treat the elements in the networks derived from Steps (1–2) as features for each subject. Then, LASSO algorithm (Tibshirani, 1996) is used to select multi-level high(er)-order FC features that are most relevant to the classification task.
4. Ensemble classification. We construct an ensemble classifier with multiple linear SVM (support vector machine) classifiers (Cortes and Vapnik, 1995); each is trained using a specific level of FC features. The classification scores by all SVM classifiers are fused by weighted averaging to produce the final classification result.

The main contribution of this paper is devising a multi-level higher-order FC representation strategy to capture the interactions among brain regions at multiple levels. As such, the features generated in different levels can contain supplementary information for joint classification.

## Materials and data preprocessing

The rs-fMRI dataset used in this study are obtained from the ABIDE database (Martino et al., 2014), which was created as a data repository for facilitating collaboration across laboratories to help accelerate scientific discovery in the autism research. To alleviate data heterogeneity, we randomly retrieved the rs-fMRI images from 54 ASD patients (47 male and 7 female) and 46 normal controls (40 male and 6 female) under 15 years of age, scanned at New York University Langone Medical Center. The detailed demographic information of the two groups, including age, gender, full-scale intelligence quotient (FIQ), and head motion (characterized by frame-wise displacement (FD)), were analyzed in Table 1. As we can see from Table 1, there were no significant differences (*p* > 0.05) in age, gender, FIQ, and FD between the normal control and ASD groups. ASD subjects were diagnosed based on the autism criteria in Diagnostic and Statistical Manual of Mental Disorders, 4th Edition, Text Revision (DSM-IV-TR) (American Psychiatric Association, 2000). More details on data collection, exclusion criteria, and scan parameters are available on the ABIDE website^2^

**Table 1:** The demographic information for ASD group and NC group.

	**Gender(M/F)**	**Age(years)**	**FIQ(mean±sd)**	**FD(mm)**
		**(mean±sd)**		**(mean±sd)**
ASD	47/7	10.7 ± 2.28	109.41 ± 18.78	0.15 ± 0.07
NC	40/6	11.22 ± 2.34	114.20 ± 12.73	0.14 ± 0.05
*p*	0.99^a^	0.27^b^	0.078^b^	0.36^b^

ASD, autism spectrum disorder; NC, normal control; M, male; F, female; FIQ, full scale intelligence quotient; FD, frame-wise displacement; p^a^: Statistical significance level was calculated using the χ^2^-test; p^b^: Statistical significance level was computed using the two-tailed two-sample t-test.)

The subjects were scanned on a 3-Tesla Siemens Allegra scanner over 6 min, producing 180 time points at a repetition time of 2 s. In Table 2, we summarize main scanning parameters used in this study. The children taking psychostimulants were required to withhold the medication at least 24 h prior to the scan and subject to physician approval. During the rs-fMRI scan, most individuals were asked to relax with their eyes open, while a white cross-hair against a black background was projected on a screen. Their eye status was monitored by an eye tracker. The mean frame-wise displacement was computed to describe head motion for each individual. The individuals were excluded if their mean FD is larger than 1 mm (Lin et al., 2015; Ray et al., 2015). On the other hand, head motion effect was further corrected with the Friston 24-parameter model in the following process.

**Table 2:** The rs-fMRI acquisition parameters.

**Parameter**	**Make(model)**	**Voxel Size**	**Flip Angle**	**TR/TE**
**Value**	Siemens Magnetom (Allegra)	3.0 × 3.0 × 4.0 (mm^3^)	90 (deg)	2,000/15 (ms)
**Parameter**	**FOV read**	**Slice thickness**	**Bandwidth**	**# of Slices**
**Value**	240 (mm)	4.0 (mm)	3906 (Hz/Px)	33

For rs-fMRI data preprocessing, we used a widely adopted Data Processing Assistant for rs-fMRI (DPARSF) toolbox (Yan and Zang, 2010). Specifically, the first 20-s data were discarded to ensure magnetization stabilization. Slice acquisition timing was corrected for each volume, followed by head motion correction (i.e., realignment) with rigid-body transformation. Then, all rs-fMRI volumes were normalized to the Montreal Neurological Institute (MNI) space and resampled to a resolution of 3 × 3 × 3 mm^3^. Data scrubbing was further carried out to reduce the negative effect of head motion, and the volumes with FD larger than 0.5 mm were removed (Power et al., 2012), along with the preceding two time points and the following two time points. We further performed the two-tailed two-sample *t*-test on the number of volumes left after scrubbing to investigate if there exist significant difference between ASD group and NC group. We got a *p*-value of 0.19. Thus, this indicated there was no significant difference (*p* > 0.05) between the two groups in term of the number of volumes. Then, white matter, cerebral spinal fluid (CSF), global signals were regressed out as nuisance covariates. Head motion was corrected with the Friston 24-parameter model (i.e., 6 head motion parameters, 6 head motion parameters from the previous time point and the 12 corresponding squared items) to regress out head motion effects from the realigned data (Satterthwaite et al., 2013; Yan et al., 2013). Next, we parcellated the brain space into 116 regions-of-interest (ROIs) by applying the Automatic Anatomical Labeling (AAL) atlas (Tzourio-Mazoyer et al., 2002) to each image. For each ROI, we computed its mean time series and performed the band-pass filtering (0.01–0.08 Hz) for trading-off between avoiding physiological noise (Cordes et al., 2001), measurement error (Achard et al., 2008), and magnetic field drifts of the scanner (Tomasi and Volkow, 2010).

## Methods

Because each FC network is represented as a fully-connected graph in a matrix format, we will mainly introduce how the corresponding matrices of the low-order and high-order FC networks are constructed in this section. Specifically, we first introduce how we derived the low-order FC network (*LON*) from the rs-fMRI time-series of a subject. Next, we introduce the construction strategy of *multi-level* high-order FC networks (*HON*s). Finally, the multi-level brain FC feature extraction, selection, and classification framework is described.

### Conventional low-order FC network construction

For each subject, we define $x_{i}\in R^{M}$ as the average rs-fMRI signal of all BOLD time-series signals in the voxels belonging to the *i*-th ROI. Here, *M* denotes the total number of temporal image volumes. We compute the Pearson's correlation between the *i*-th and the *j*-th ROIs as follows:

(1) $$\begin{array}{r} c_{ij}=corr\left(x_{i},x_{j}\right) \end{array}$$

Then, a conventional correlation-based FC network (i.e., *low-order* FC network) is generated by a corresponding symmetric matrix *C*_*LON*_, as defined below:

(2) $$\begin{array}{r} C_{LON}={\left(c_{ij}\right)}_{1\leq i,j\leq M} \end{array}$$

where each row or column of *C*_*LON*_ denotes the Pearson correlation series between a specific ROI and all other ROIs. Each element in *C*_*LON*_ is the Pearson correlation between the average time-series of a pair of ROIs *i* and *j*. Notably, *C*_*LON*_ encodes low-order interactions between any pair of ROIs.

### Multi-level high-order FC networks construction

To fully capture high-order functional interactions across brain regions, we adopt a method proposed in (Zhang et al., 2016, 2017a) to generate the high-order FC networks based on “correlation's correlation.” Specifically, let *c*_*i*_ = (*c*_*i*1_, *c*_*i*2_, … , *c*_*iM*_) denote a vector containing the correlations between the *i*-th ROI and all other ROIs. Mathematically, *c*_*i*_ denotes the *i*-th row or column of the symmetric matrix *C*_*LON*_ in Equation 2. We compute the “correlation's correlation” between the *i*-th ROI and the *j*-th ROI as follows:

(3) $$\begin{array}{r} c_{ij}^{2}=\;corr\left(c_{i},c_{j}\right) \end{array}$$

where *c*_*i*_ = (*c*_*i*1_, … , *c*_*i*(*i*−1)_, *c*_*i*(*i*+1)_, … , *c*_*i*(*j*−1)_, *c*_*i*(*j*+1)_, … , *c*_*iM*_) and *c*_*j*_ = (*c*_*j*1_, … , *c*_*j*(*i*−1)_, *c*_*j*(*i*+1)_, … , *c*_*j*(*j*−1)_, *c*_*j*(*j*+1)_, … , *c*_*iM*_). $c_{ij}^{2}$ indicates how the FC profiles between the *i*-th ROI and all other ROIs *resemble* the FC profiles between the *j*-th ROI and all other ROIs, which can reveal more complex relationship between the FC profiles (or the vectors {*c*_*i*_}), not just the original rs-fMRI time series *x*_*i*_. As a result, the correlation $c_{ij}^{2}$ in Equation 3 can extract interaction information from all different ROIs, whereas the correlation *c*_*ij*_ in Equation 1 involves just the two different ROIs. In other words, the correlation coefficient $c_{ij}^{2}$ is able to characterize more complex and abstract interaction among multiple brain regions. Thus, the corresponding matrix *C*_*HON*−1_ of the first-level of high-order FC network (*HON-1*) can be defined as follows:

(4) $$\begin{array}{r} C_{HON-1}={\left(c_{ij}^{2}\right)}_{1\leq i,j\leq M} \end{array}$$

Furthermore, for a specific subject, we can obtain multi-level FC networks by their corresponding matrix series, i.e., {*C*_*LON*_, *C*_*HON*−1_, … , *C*_*HON*−*t*_}, in a subsequent level-by-level manner, in which each matrix *C*_*HON*−*i*_ (*i* ≥ 2) is derived from the previous-level matrix *C*_*HON*−(*i*−1)_. In this way, higher-level connectivity features can be obtained from the low-level connectivity features, and thus form hierarchical representations of functional interactions across multiple brain regions.

### Multi-level FC feature extraction, selection and classification

For the *l*-th subject, we use its corresponding set of multi-level FC matrices $\{C_{LON}^{\left(l\right)},C_{HON-1}^{\left(l\right)},\ldots ,C_{HON-t}^{\left(l\right)}\}\;$as raw features. Noting the symmetry of each FC matrix, we only vectorize its lower off-diagonal triangular part to define the feature vectors, i.e., $\left\{y_{0}^{\left(l\right)},y_{1}^{\left(l\right)},\ldots ,y_{t}^{\left(l\right)}\right\}$, for representing the *l*-th subject. The dimensionality of $y_{i}^{\left(l\right)}\;\left(0\leq i\leq t\right)$ is $\frac{\;M\left(M-1\right)}{2}$, where *M* denotes the number of ROIs as mentioned above.

The feature vectors $\left\{y_{0}^{\left(l\right)},y_{1}^{\left(l\right)},\ldots ,y_{t}^{\left(l\right)}\right\}$ extracted from multi-level FC networks might include irrelevant or redundant features for ASD diagnosis. Therefore, feature selection is necessary. In order to select a small subset of features that are most relevant to ASD pathology, we adopt *L*_1_-norm regularized least squares regression, known as LASSO (Least Absolute Shrinkage and Selection Operator) (Tibshirani, 1996), due to its simplicity and efficiency (Wee et al., 2012, 2016; Jin et al., 2015; Biao et al., 2016). Specifically, let $\omega_{i}={\left(w_{i1},w_{i2},\ldots \;,w_{id}\right)}^{T}$ represent the weight vector for the feature selection task and $K={\left(k_{1},k_{2},\ldots \;,k_{N}\right)}^{T}$ is the class labels of *N* training data (from *N* training subjects). Here, *d* is the number of features. Mathematically, the LASSO model can be described as follows:

(5) $$\frac{1}{2}\sum_{l=1}^{N}{\left\|k_{l}-{\left(y_{i}^{\left(l\right)}\right)}^{T}\omega_{i}\right\|}_{2}^{2}+\lambda {\left\|\omega_{i}\right\|}_{1}$$

where λ is a parameter for controlling the strength of *L*_1_-norm regularization. The first term in Equation 5 is the empirical loss on the training data, and the second term is the *L*_1_ − *norm* regularization term that is used to enforce some elements of ω_*i*_ to be zero (i.e., corresponding to non-discriminative features in our classification task). In this way, we can jointly achieve classification error minimization and sparse feature selection. Let$\left\{{\tilde{y}}_{0}^{\left(l\right)},{\tilde{y}}_{1}^{\left(l\right)},\ldots ,{\tilde{y}}_{t}^{\left(l\right)}\right\}$ denote selected features from the original feature vectors $\left\{y_{0}^{\left(l\right)},y_{1}^{\left(l\right)},\ldots ,y_{t}^{\left(l\right)}\right\}$.

After selecting the most important features by LASSO, we use SVM with a linear kernel for ASD classification (Cortes and Vapnik, 1995). SVM seeks a maximum margin hyperplane to separate the samples of one class from another class. The empirical risk on training data and the complexity of the model can be balanced by the hyper-parameter γ, thus ensuring good generalization ability on the unseen data. Herein, we train an ensemble of *L* SVM classifiers, each trained on a specific feature set ${\left\{{ỹ}_{i}^{\left(l\right)}\right\}}_{l=1}^{L}\left(i=0,1,\ldots \;,t\right)$, where *L* denotes the number of levels used for computing different levels of functional connectivity. Then, the decision scores from all SVM models are fused linearly (by a weighting parameter **α** tuned for each SVM, α was selected from 0.1 to 0.9 with step 0.1) to produce the final label for the target subject. Note that we use 10-fold cross validation on the training data to evaluate the performance **α** of our algorithm for fair comparison. Hence, the value of α might change across cross-validated folds.

## Experiments

For evaluation, we tested our proposed method for classifying ASD and NC subjects. We also performed feature weight analysis to identify multi-level brain connections that are most discriminative for classifying ASD and NC.

### Comparison of ASD diagnosis using different feature types

For comparison, we used connectional brain features extracted from different orders of FC networks, including the matrix *C*_*LON*_ from *LON*, *C*_*HON*−1_ from *HON-1*, *C*_*HON*−2_ from *HON-2*, and their combinations. We trained a set of linear SVMs based on the LIBSVM toolbox^3^, each using a set of specific-level connectional features. For the case using specific-level connectional features, the output of each SVM is regarded as the final classification result. For the case of using the combination of different levels of connectional features, the final classification result is obtained by fusing decision scores from all SVMs.

In this study, we adopted a 10-fold cross-validation strategy to evaluate the generalization performance of our proposed method. Basically, all training subjects were partitioned into 10 subsets (each subset with a roughly equal sample size), and each time the samples within one subset are selected as the testing dataset, while the remaining samples in the other 9 subsets are combined together as the training dataset for feature selection and classification. Finally, we report the average accuracy of classification results across all 10 cross-validation folds.

As the performance of our method depends on a few hyper-parameters, such as **λ** in the feature selection step (see Equation 5), **γ** in SVM model, and **α** in the decision fusion step, it is important to fine-tune these hyper-parameters. Hence, we used a nested cross-validation on the training data to automatically identify the optimal values for these hyper-parameters within the following ranges: **λ** ∈ [**0**.**1**, **0**.**2**, … , **0**.**6**], **γ** ∈ [**2**^−**5**^, **2**^−**4**^, … , **2**^**5**^], and **α** ∈ [**0**.**1**, **0**.**2**, … , **0**.**9**]. Specifically, we further split the training set into the training subset and the validation subset and further performed another cross-validation. That is, for each combination of values for hyper-parameters, the validation subset from the training set is used for testing and the remaining training subset is used for training. This procedure was repeated 10 times, which produced a classification accuracy under a specific combination of hyper-parameter values. Then, the hyper-parameter values with the best classification accuracy on the validation data were chosen and used to construct the optimal model based on all the training samples. The constructed model with optimized parameters was applied to the testing data.

For comprehensive evaluations, we used six different statistical measures, namely classification accuracy (ACC), sensitivity or true positive rate (TPR), specificity or true negative rate (TNR), precision or positive predictive value (PPV), negative predictive value (NPV), and F1 score^4^ Higher values for these scores indicate better performance.

To avoid biased results due to the fold selection, the entire 10-fold cross-validation process was further repeated 20 times, each with a different partition of subjects. The average statistics of the 20 repetitions were finally reported. Table 3 shows the mean classification performance for each compared feature type, where *C*_*LON*_ denotes the feature derived from the low-order FC networks (*LON*) and *C*_*LON*_+*C*_*HON*−1_ denotes the combination of *LON* and *HON-1*. The meaning of the other symbols is similar. We also use the bold font to highlight the best results in the Table 3. At the same time, each feature type was also given a serial number for simplifying its description in the following. In order to investigate if there is any significant difference in ASD classification when different feature types were used, we performed the pair-wise *t*-test based on the 10-fold cross-validation accuracies. The *p* values at the 5% significance level are reported in Table 4, where each serial number denotes a different model using corresponding feature type in Table 3. The *p* values between the feature derived from *C*_*LON*_ + *C*_*HON*−1_ and any other feature type are highlighted in bold.

**Table 3:** ASD classification using different feature types.

	**Feature type**	**ACC**	**TPR**	**TNR**	**PPV**	**NPV**	**F1**
1	*C*_*LON*_	0.73	0.75	0.70	0.74	0.72	0.75
2	*C*_*HON*−1_	0.70	0.73	0.67	0.70	0.70	0.71
3	*C*_*HON*−2_	0.67	0.74	0.64	0.65	0.74	0.69
4	*C*_*LON*_+*C*_*HON*−1_	**0.81**	**0.82**	**0.80**	**0.83**	**0.78**	**0.83**
5	*C*_*LON*_+*C*_*HON*−2_	0.76	0.77	0.75	0.80	0.72	0.78
6	*C*_*HON*−1_+*C*_*HON*−2_	0.72	0.77	0.67	0.69	0.76	0.73
7	*C*_*LON*_+*C*_*HON*−1_+*C*_*HON*−2_	0.78	0.81	0.75	0.78	**0.78**	0.79

**Table 4:** Significance test between different pair of feature types.

	**2**	**3**	**4**	**5**	**6**	**7**
**1**	0.044	0.037	**0.018**	0.047	0.049	0.040
**2**		0.042	**0.003**	0.025	0.042	0.024
**3**			**0.001**	0.034	0.046	0.03
**4**				**0.036**	**0.024**	**0.047**
**5**					0.034	0.049
**6**						0.045

As we can see from Table 4, there were significant differences (*p* < 0.05) in classification performance between any two different feature types. It indicates that the *C*_*LON*_ + *C*_*HON*−1_ method significantly outperforms all other methods. From the results shown in Table 3, we can draw the following conclusions. (1) Compared with the single feature types, the combination of functional features with different orders can achieve better diagnostic accuracy. This indicates that different feature types can provide complementary information for diagnosis. (2) The combination of *C*_*LON*_ and *C*_*HON*−1_ achieves the best performance for all metrics, which might indicate that there exists more strongly complementary information between *LON* and *HON*−1. In contrast, other combinations of different feature types possibly include more irrelevant or redundant information, thus affecting their discriminative performance in classification.

In addition to the above ensemble learning for integrating low-order and high-order networks, we also evaluate another widely adopted strategy by firstly concatenating the features from different FC networks and then performing feature selection with LASSO and constructing a single linear SVM classification. The experimental results are shown in Table 5, where ⊕ denotes simple feature concatenation. For example, *C*_*LON*_ ⊕ *C*_*HON*−1_ denotes the concatenated features from the *LON* and *HON-1*. As we can see, simply concatenating the features from different types of networks only slightly improves the classification performance when compared to those using single type of brain networks (using either *LON* or *HON-1*), but is inferior to the ensemble classification (Table 3). The possible reason of such results is that considering different FC networks may contain information at different levels, leading to different distributions of their corresponding features. Simply concatenating the features can make the feature correlation and distribution more complex, making it difficult to capture by the traditional feature selection methods. In contrast, constructing two classifiers in respective feature space is able to avoid this problem and thus provide more reliable results.

**Table 5:** Classification accuracy based on simple feature concatenation.

**Feature type**	**ACC**	**TPR**	**TNR**	**PPV**	**NPV**	**F1**
*C*_*LON*_ ⊕ *C*_*HON*−1_	0.79	0.81	0.77	0.80	0.77	0.80
*C*_*LON*_ ⊕ *C*_*HON*−2_	0.74	0.79	0.69	0.70	0.78	0.75
*C*_*HON*−1_ ⊕ *C*_*HON*−2_	0.72	0.74	0.70	0.74	0.70	0.74
*C*_*LON*_ ⊕ *C*_*HON*−1_ ⊕ *C*_*HON*−2_	0.77	0.79	0.74	0.78	0.76	0.79

### The most discriminative features for ASD diagnosis

Based on the results of LASSO regression, we identified the most discriminative low-order and high-order functional features as those with the highest selection frequency across all 10-fold cross-validation runs. Note here we used the frequency of a feature to be selected in all cross-validation runs to reflect the contribution of the feature to the classification. Higher frequency indicates a larger contribution of the corresponding feature.

**Figure 2:**
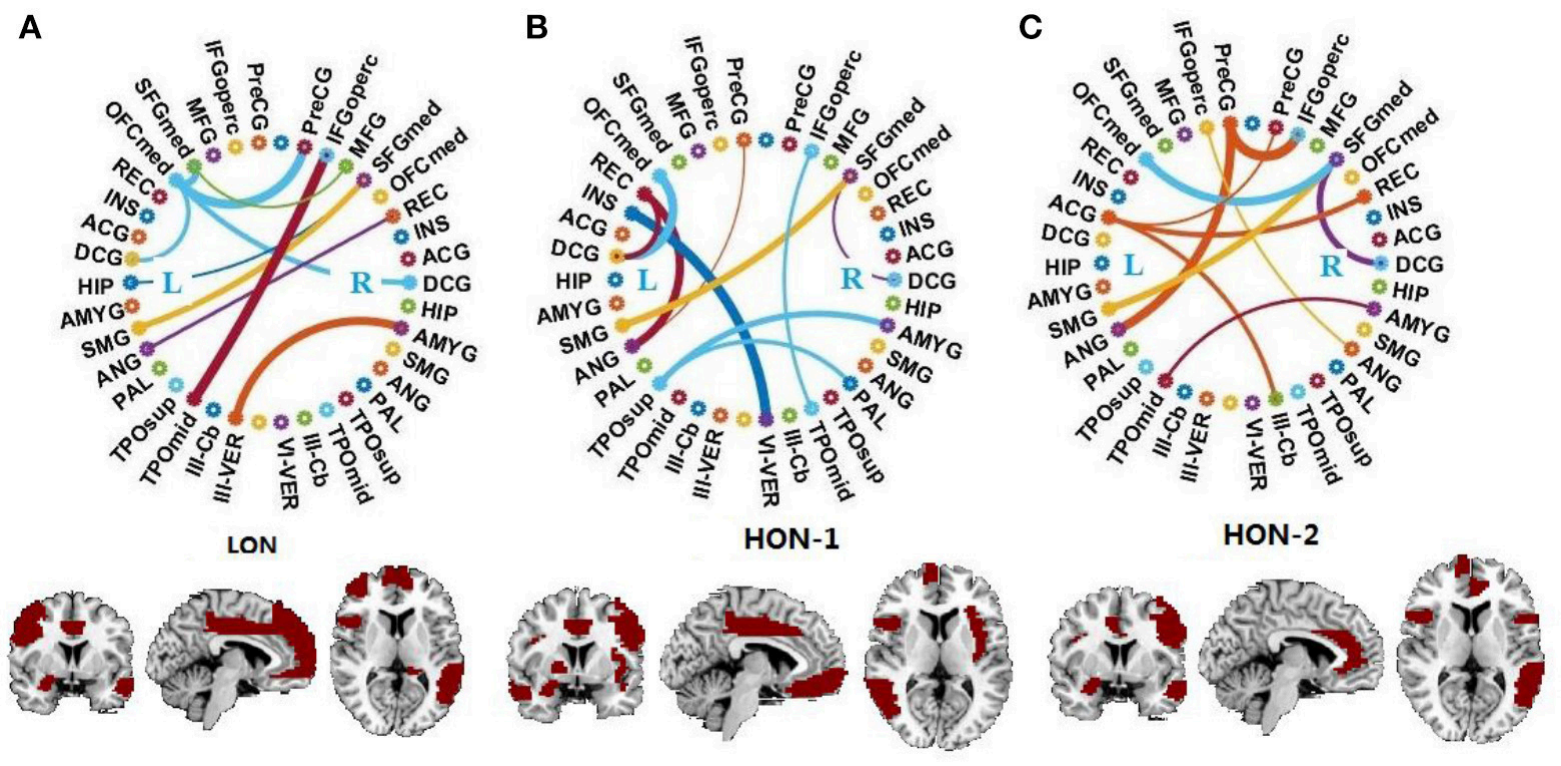
Connectogram and involved brain regions of the top 10 discriminative connections selected by our framework in **(A)** the low-order FC network (*LON*), **(B)** the first-level high-order FC network *(HON-1*) and **(C)** the second-level high-order FC network *(HON-2*), respectively. The thickness of each line reflects its selection frequency, i.e., thicker lines indicate higher selection frequency. The brain slice view shows the involved brain regions (or ROIs). The brain slices were located at (−5, 4, 9) in the standard Montreal Neurological Institute (MNI) space. For the abbreviations of brain regions, please refer to Table 6.

Figure 2 displays the connectogram of the 10 most discriminative connections, where each connection denotes the correlation between two brain regions (Krzywinski et al., 2009). The thickness of each line reflects the frequency of being selected for the respective feature, i.e., a thicker line indicating higher frequency of being selected in all cross-validation runs. We also list the abbreviations of the selected ROIs in Table 6, and use the bold font to highlight the ROIs that are related to the perception of emotion, the interpretation of sensory information, language performance, and sports coordination (Herbert et al., 2005; Krzywinski et al., 2009; Ha et al., 2015).

**Table 6:** ROIs selected from *LON, HON-1*, and *HON-2*.

**Abbreviation**	**ROI name**	**Abbreviation**	**ROI name**
PreCG	Precentral gyrus	**IFGoperc**	**Inferior frontal gyrus (opercula)**
**MFG**	**Middle frontal gyrus**	SFGmed	Superior frontal gyrus (medial)
**OFCmed**	**Orbitofrontal cortex (medial)**	REC	Rectus gyrus
**INS**	**Insula**	**ACG**	**Anterior cingulate gyrus**
DCG	Middle cingulate gyrus	**HIP**	**Hippocampus**
**AMYG**	**Amygdala**	**SMG**	**Supramarginal gyrus**
**ANG**	**Angular gyrus**	**PAL**	**Pallidum**
**TPOsup**	**Temporal pole (superior)**	TPOmid	Temporal pole (middle)
VI-VER	Lobule VI of vermis	III-VER	Lobule III of vermis
III-Cb	Lobule III of cerebellar hemisphere		

From the results shown in Figure 2 and Table 6, we derive the following conclusions. (1) It can be clearly observed that the discriminative connections and brain regions are distributed across both hemispheres and different lobes, indicating the distributed pattern of functional abnormalities over the whole brains of ASD patients. (2) The majority of brain regions with top selection frequencies, such as inferior frontal gyrus, amygdala, angular gyrus, and hippocampus, are related to social communication, emotion expression, language comprehension, and action coordination (Herbert et al., 2005; Ecker et al., 2015; Ha et al., 2015). These findings are in agreement with the behavioral phenotype of ASD (Geschwind and Levitt, 2007; American Psychiatric Association, 2013). (3) The selected features from the high-order network are largely different from those from the low-order network, indicating that different functional networks may provide complementary discriminative information for diagnosis.

## Conclusion

In this article, we proposed extracting multi-level high-order FC networks, derived from rs-fMRI, to capture the high-order correlation across different brain regions for ASD diagnosis. This is based on our hypothesis that different pairs of brain regions could influence each other, and their high-order correlations could contain more important discriminative information for ASD diagnosis, which is actually consistent with previous works, i.e., in Chen et al. (2016), Wee et al. (2016), Zhang et al. (2016, 2017a,b), Zhou et al. (2018). This important high-order connectivity information is overlooked in most existing methods for ASD diagnosis, which simply focused on low-order correlations between pairs of brain regions.

Experimental results have shown that (1) high-order FC networks indeed include crucial discriminant information for ASD diagnosis, and (2) the combination of different order FC networks, especially *LON* and *HON-1*, can significantly improve ASD diagnostic performance. Furthermore, we found that the most discriminative brain regions are related to episodic memory, social cognition and emotion processing. These findings are in line with the behavioral phenotype of ASD, which is associated with several impairments of interaction, language, behavior, and cognitive functions.

Lastly, it should be noted that we used a simple feature selection method, thus the selected features may still include redundant information, which could affect our classification accuracy. Accordingly, the strategies for discriminative feature selection and fusion need further investigation, which will be investigated in our future work. In addition, it should be noted that LASSO regression tends to select only one feature from multiple highly correlated features. In the context of diagnosis, this means that, although these features could be also essentially valuable for discrimination, they might be discarded after feature selection due to the multi-collinearity in the data matrix. In this work, we mainly followed the lead of previous studies (Jin et al., 2015; Biao et al., 2016; Wee et al., 2016) and applied LASSO to select features since it has shown many merits in reducing model dimensionality and ameliorating overfitting problem. In our future work, the other features that might have been discarded but are highly correlated with those selected ones deserve dedicated investigation.

## Author contributions

All authors listed have made a substantial, direct and intellectual contribution to the work, and approved it for publication.

### Conflict of interest statement

The authors declare that the research was conducted in the absence of any commercial or financial relationships that could be construed as a potential conflict of interest.

## Footnotes

^1^https://www.cdc.gov/ncbddd/autism/data.html.

^2^http://fcon_1000.projects.nitrc.org/indi/abide/abide_I.html.

^3^https://www.csie.ntu.edu.tw/~cjlin/libsvm/.

^4^https://en.wikipedia.org/wiki/Sensitivity_and_specificity.
